# Molecular Mechanisms of Kaposi Sarcoma-Associated Herpesvirus (HHV8)-Related Lymphomagenesis

**DOI:** 10.3390/cancers16213693

**Published:** 2024-10-31

**Authors:** Caroline J. Yu, Blossom Damania

**Affiliations:** Department of Microbiology & Immunology, Lineberger Comprehensive Cancer Center, University of North Carolina, Chapel Hill, NC 27599, USA; cjyu@unc.edu

**Keywords:** Kaposi sarcoma-associated herpesvirus, KSHV, HHV8, lymphoma, lymphomagenesis, viral oncogenesis

## Abstract

Approximately 15–20% of cancers are caused by viruses. Kaposi sarcoma-associated herpesvirus (KSHV), also known as human herpesvirus 8 (HHV8), is an oncogenic virus that is the etiologic agent of not only Kaposi sarcoma but also the lymphoproliferative disorders, primary effusion lymphoma (PEL) and multicentric Castleman disease (MCD). KSHV can infect a broad tropism of cells, including B lymphocytes, wherein KSHV encodes specific viral proteins that can transform the cell. KSHV infection precedes the progression of PEL and MCD. KSHV establishes lifelong infection and has two phases of its lifecycle: latent and lytic. During the latent phase, viral genomes are maintained episomally with limited gene expression. Upon sporadic reactivation, the virus enters its replicative lytic phase to produce infectious virions. KSHV relies on its viral products to modulate host factors to evade immune detection or to co-opt their function for KSHV persistence. These manipulations dysregulate normal cell pathways to ensure cell survival and inhibit antiviral immune responses, which in turn, contribute to KSHV-associated malignancies. Here, we highlight the known molecular mechanisms of KSHV that promote lymphomagenesis and how these findings identify potential therapeutic targets for KSHV-associated lymphomas.

## Simple Summary

Kaposi sarcoma-associated herpesvirus (KSHV) is associated with lymphoproliferative disorders, including primary effusion lymphoma and multicentric Castleman disease. KSHV expresses viral proteins that aid in the evasion of antiviral immune responses and manipulate host factors and signaling to promote cell survival. By altering the cell environment, KSHV contributes to lymphomagenesis.

## 1. Introduction

Kaposi sarcoma-associated herpesvirus (KSHV) is a member of the gamma-herpesvirus family, a subgroup of herpesviruses (including Epstein–Barr virus) with oncogenic potential. KSHV is a large double-stranded DNA virus with a 165 kb genome encoding over 100 genes. KSHV is associated with three malignancies, Kaposi sarcoma (KS) and two lymphoproliferative disorders, including primary effusion lymphoma (PEL) and multicentric Castleman disease (MCD). PEL and MCD are B cell lymphoproliferative diseases.

KSHV seroprevalence is dependent on the geographic region of the world. Seroprevalence is greater than 30% in sub-Saharan Africa, 15–20% in the Mediterranean region, and 2–5% in other regions around the world [1]. KSHV was first detected in a KS lesion from an acquired immunodeficiency syndrome (AIDS) patient [2]. Subsequently, KSHV was also detected in tissues from PEL and MCD patients [3,4].

KSHV establishes lifelong infection. Like all herpesviruses, KSHV has a biphasic life cycle with latent and lytic phases. During the latent phase, viral genomes are maintained episomally with limited gene expression. Latent genes help maintain viral DNA and prevent apoptosis, ensuring KSHV persistence. Upon sporadic reactivation, the virus enters its replicative lytic phase with elevated viral gene expression. Reactivation can be triggered by environmental stimuli such as stress and UV radiation, to name a few. Lytic replication allows for the temporally orchestrated expression of immediate early (IE), early (E), and late (L) viral genes for structural proteins to produce infectious virions (reviewed in [5]).

KSHV modulates host factors to evade immune detection or to co-opt their function for KSHV persistence. These manipulations dysregulate typical cell processes to ensure cell survival and inhibit antiviral immune responses, which, in turn, contribute to KSHV-associated malignancies. Both latent and lytic genes contribute to oncogenesis. Latent genes are continuously expressed in the tumor cell, and a small percentage of cells undergo lytic reactivation, where lytic proteins are thought to condition the microenvironment to promote KSHV-associated tumorigenesis (reviewed in [6]).

KSHV can infect a broad tropism of cells (reviewed in [7]), including B lymphocytes. B cells are the cell type of origin for both PEL and MCD. Here, we provide an overview of KSHV lymphoproliferative diseases and highlight the known molecular mechanisms of KSHV viral products that promote lymphomagenesis, and we explore how these findings identify potential therapeutic targets for KSHV-associated lymphomas.

## 2. Non-Hodgkin Lymphoma

Lymphomas are divided into Hodgkin or nonHodgkin lymphomas. Non-Hodgkin lymphoma (NHL) is the seventh most prevalent cancer and sixth most common cause of cancer-related death in the United States [8].

Non-Hodgkin lymphomas occur when lymphocytes grow abnormally and accumulate to form tumors. NHLs are a heterogeneous group of lymphoproliferative malignancies with over 60 subtypes, and the subtypes are further categorized by disease progression, either indolent (slow) or aggressive (fast). NHL subtypes mostly comprise diffuse large B cell lymphomas (~30%) and follicular lymphomas (~20%), while all other subtypes are <10% [9]. A total of 85–90% of NHL cases originate in B cells [10]. The most recent classification developed by the World Health Organization classifies types of NHL by type of lymphocyte (B, T, or NK cell), by the location the lymphoma originates from, and by the features of the cells, such as their morphology, specific surface protein markers, and genetic features of chromosomes [11].

The biggest risk factors for NHL are immunosuppression, UV radiation, viral infection, autoimmune and chronic inflammatory disorders, and occupational exposures [12]. Infections that weaken the immune system or cause chronic immune stimulation increase the risk. Chronic antigenic stimulation can also increase B cell proliferation, thus increasing the likelihood of mutations, especially immunoglobulin rearrangements. The cause of lymphomagenesis can be related to mutations in oncogenes, tumor suppressor genes, DNA repair genes, or viral infection.

### Primary Effusion Lymphoma

Primary effusion lymphoma is a highly aggressive NHL with a poor prognosis, a 6-month median survival post diagnosis, and very limited efficacious therapeutics. PEL is rare, and it is primarily seen in HIV-positive individuals; however, KSHV is the causative agent [13]. PEL cells are predominantly latently infected with KSHV. PELs are B-cell-derived lymphomas that primarily develop in body cavities like the pleural, peritoneal, and pericardial spaces [14]. PEL patients show symptoms from fluid accumulation, such as difficulty with breathing, abdominal distension, and chest pain. PEL cells are large and anaplastic with irregular nuclei and prominent nucleoli. Based on VDJ arrangements, PEL cells are clonal in nature and express some B cell markers, including CD45, CD30, CD138, and other activation markers that resemble partially differentiated post-germinal center B cells, but they are CD20-negative [13]. Pleural and peritoneal effusions from patients with PEL generally contain high concentrations of viral IL-6 (vIL-6), as well as human IL-6 (hIL-6) and IL-10 [15]. These cytokines promote the autocrine growth of PEL cells.

KSHV is the etiologic agent of PEL, where 100% of PEL cells contain KSHV genomes. However, approximately 80% of PELs are co-infected with the related gamma-herpesvirus, Epstein–Barr virus (EBV) (reviewed in [16]). Both viruses typically persist in cells in their latent, quiescent state, with their viral genomes tethered to host chromatin as episomes, implying there may be a fitness advantage for the cell to retain these viruses. A selection of KSHV genes are known to be critical to the survival or proliferation of PEL cells, so KSHV is considered a genetic driver of PEL [17,18]. KSHV viral genes have been shown to be important for PEL survival, and the depletion of viral genes, e.g., KSHV *vFLIP*, *vCyclin*, and *vIRF3*, causes apoptosis of PEL and inhibits PEL growth in immunodeficient mice [17,18].

When EBV infection is also present in PEL, EBV latency I pattern is observed, where EBNA-1, EBER RNA, and low levels of LMP2a are expressed [19,20]. A study showed that co-infection with EBV allows for KSHV persistence in humanized mice, resulting in PEL-like lymphomagenesis [21]. EBV supports KSHV persistence after primary B cell infection and improves KSHV DNA maintenance after infection of EBV-negative PEL in vitro [22]. KSHV and EBV influence each other, where KSHV has been shown to enhance EBV lytic replication. Increased EBV lytic infection contributes to more frequent lymphomagenesis, which has been observed in both doubly infected B cells of humanized mice and co-infected human PEL cells [23,24]. Another study defined conditions to stably infect human B cells with KSHV and showed that optimal infection required coinfection by EBV [25]. Dually infected B cells were stably transformed, with the maintenance of both EBV and KSHV.

## 3. Multicentric Castleman Disease

Half of multicentric Castleman disease (MCD) cases are associated with KSHV, while the other half are idiopathic [26]. MCD is a lymphoproliferative disorder and is characterized by lymphadenopathy with polyclonal hypergammaglobulinemia and high serum levels of IL-6. KSHV-associated MCD is distinct from other forms of MCD, with a more aggressive clinical presentation compared to idiopathic MCD [27,28]. In 1995, KSHV was discovered as the etiologic agent of a plasmablastic variant of MCD occurring most commonly in HIV-infected patients (90%). MCD in HIV-infected patients usually develops 3 years after HIV diagnosis and is commonly identified alongside KS [29]. In HIV-negative patients, KSHV-MCD is largely seen in populations with a high prevalence of KSHV infection, such as men who have sex with men and populations from sub-Saharan Africa, where KSHV is endemic [30,31]. A minority of KSHV-infected individuals develop MCD years later, indicating the relevance of cofactors in the development of disease. Additionally, patients with MCD are more susceptible to other NHLs and organ failure [26].

Plasmablasts in MCD have an abnormal proliferation of immunoglobulin-restricted naïve B cells in the B cell mantle zone and interfollicular regions [13]. In KSHV-MCD, both viral and human IL-6 contribute to the plasmacytic differentiation of these cells, and high levels of vIL-6 are detected in the serum of MCD patients [32,33]. At times, both KS and KSHV-MCD can be observed in the same lymph node; however, plasmablasts are rarely coinfected with EBV [34]. While the expansion of clusters of plasmablasts can occur, these lesions are not clonal and do not always progress to lymphoma [26].

IL-6 is regarded as the driver of the proinflammatory state of MCD. It has been proposed that both nodal and systemic manifestations are reactive changes to elevated levels of IL-6 and other circulating cytokines and chemokines. Additionally, KSHV encodes a viral homolog of IL-6, vIL-6, with optimized IL-6 functions. vIL-6 has been validated as a driver in the development of MCD-like disease in mouse models [35,36]. Patients with MCD have prominent inflammatory manifestations that are attributed to vIL-6, hIL-6, and IL-10 cytokine levels. In MCD, the affected lymph nodes contain vIL-6-positive cells, and flares of the disease correlate with vIL-6 levels [37].

## 4. Mechanisms of KSHV-Associated Lymphomagenesis

### 4.1. KSHV Manipulates Host Machinery in B Cells

The molecular pathogenesis of B cell lymphomas includes aberrant intracellular signaling, disordered transcriptional and epigenetic regulation, and immune evasion [38]. B cells receive signals through receptors to induce transcriptional activity; thus, transcription factors are critical in B cell responses and are exploited by KSHV to reprogram the cell for survival. Commonly affected pathways include the NF-κB, PI3K/Akt/mTOR, and JAK/STAT pathways. KSHV is known to hijack these pathways [39,40,41]. Constitutive activation of NF-κB signaling often occurs, promoting survival, where in the context of lymphomas, negative regulators of NF-κB are often rendered inoperative [39].

Despite multiple cell cycle checkpoints, KSHV promotes the proliferation of cancer cells. Tumors form by overcoming replicative senescence. Throughout cell divisions, the telomere length of primary cells shortens. Shortened telomeres eventually activate the DNA damage response and tumor suppressor proteins p53 and retinoblastoma protein (Rb). Tumor suppressor proteins induce senescence, such that cells halt cell division but are metabolically active. For primary human cells to transform, both p53 and Rb must be inactivated. KSHV uses viral gene products and manipulates host machinery to inactivate these tumor suppressor proteins [42,43]. To sustain uncontrolled proliferation, cancer cells reprogram metabolic pathways. KSHV reprograms the cell to support its persistence and proliferation, including glucose metabolism, where KSHV-infected B and PEL cells have increased glucose uptake, increased lactate secretion, and decreased oxygen consumption. KSHV also upregulates glycolysis, promotes fatty acid synthesis, and alters metabolite sensing [44]. KSHV targets tumor suppressors and metabolic programs to funnel resources and support the continued growth of KSHV-infected cells.

KSHV infection is known to activate innate immune responses through both RNA and DNA sensors like toll-like receptors (TLRs), RIG-I-like receptors (RLRs), AIM2-like receptors (ALRs), and cyclic GMP-AMP synthase (cGAS) [45,46,47,48,49,50,51]. Once a foreign pathogen like KSHV is detected, the host innate immune response typically induces the production of type I interferons, priming cells to an antiviral state. However, KSHV evades immune detection by subverting these immune responses. The primary method of KSHV persistence is by defaulting to its latent state. Due to the low level of latent viral gene expression, the immune system may not be able to detect KSHV. KSHV also encodes viral products and co-opts cellular proteins as proviral factors to evade detection. For example, KSHV encodes a viral deubiquitinase, ORF64, which inhibits RLR-induced interferon production [52]. KSHV also uses cellular adenosine deaminase acting on RNA 1 (ADAR1) to further dampen RLR-dependent innate immune responses [53]. KSHV encodes a virion tegument protein, ORF52, LANA, and viral interform regulatory factor (vIRF1) to prevent cGAS-STING sensing [50,51,54]. Furthermore, KSHV uses cellular barrier-to-autointegration factor 1 (BAF) to compete with cGAS to prevent the cGAS-STING-mediated type I interferon response [55]. By counteracting antiviral immune responses, KSHV can persist and continue to disrupt cell function, contributing to the development of tumorigenesis.

Thus, KSHV manipulates canonical cell survival and proliferation pathways, reprograms metabolic activity, evades immune detection, and contributes to lymphomagenesis.

### 4.2. KSHV Latency-Associated Nuclear Antigen (LANA) Supports Lymphomagenesis

**Figure 1:**
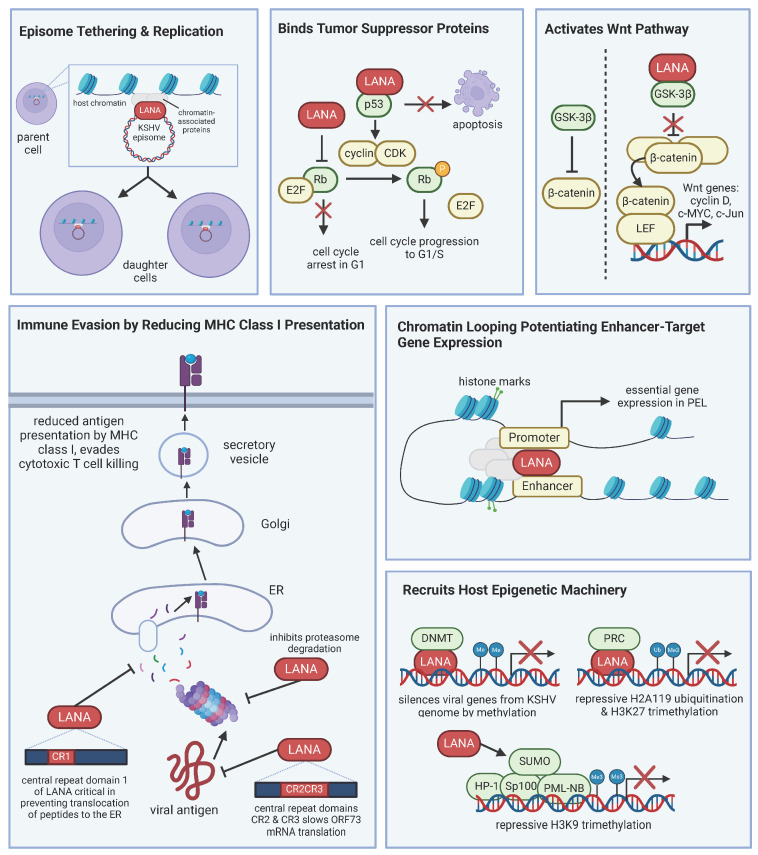
LANA manipulates host machinery for viral persistence. LANA is the master regulator of latency. LANA interacts with host proteins and has numerous functions, including, but not limited to, episome tethering for viral genome maintenance, inhibiting tumor suppressor proteins, activating pathways like Wnt, immune evasion, and controlling gene expression. The various roles of LANA contribute to KSHV-associated lymphomagenesis.

Viral oncogenes work together to promote cell survival and proliferation, creating a cell environment conducive to both KSHV persistence and lymphomagenesis. KSHV LANA is expressed in all latently infected cells and is a universal marker of KSHV infection, as it is present in KS spindle cells and MCD and PEL B cells. LANA is considered the master regulator of KSHV latency and relies on recruiting and manipulating host machinery. LANA has many functions, including maintenance of the viral genome, inhibition of tumor suppressors, activation of cellular signaling pathways to promote cell survival and proliferation, modulation of host immune responses, epigenetic regulation, and interaction with cellular proteins (Figure 1) [56].

LANA localizes to the nucleus and tethers the KSHV episome to host cell chromosomes [57], ensuring that the viral genome is replicated in tandem with host DNA during normal cell division. LANA both binds to the sequences in the terminal repeats of the KSHV genome and the mitotic chromosomes. Additionally, LANA interacts with host chromatin and replication machinery to ensure that the genome is replicated and segregated appropriately. Daughter cells stably inherit the KSHV genome, which is essential for maintaining latency. Beyond tethering, a recent analysis profiling the genome-wide occupancy of LANA and epigenomic landscape of PEL cells found that LANA participates in chromatin looping and potentiates enhancer target gene expression [58]. The study observed increased levels of enhancer RNA transcription and activated chromatin marks at LANA-bound enhancers. The study examined LANA-occupied enhancers target genes and found that the identified targets are genes known to be critical for latency and tumorigenesis.

LANA also binds tumor suppressor proteins p53 and Rb [42,43]. When LANA binds p53, it inhibits p53’s transcriptional activity and prevents p53-mediated apoptosis [42,59]. Infected cells can then evade cell cycle arrest and apoptosis, leading to uncontrolled cell proliferation. LANA interacts with Rb, which dysregulates Rb-mediated control of the cell cycle, leading to unrestrained cell division [43]. The full contribution of LANA binding Rb in the context of PEL is not known because while a proportion of Rb complexes to LANA, PEL cell lines show a lack of cyclin D-CDK inhibitor, p16INK4a, and BC3 PEL cells lack Rb altogether [60]. PEL cells remain sensitive to growth inhibition when p16INK4a expression is restored, implying that either Rb function in PEL cells is not completely inactive or that KSHV-infected cells are resistant to p16INK4a. Thus, LANA may only partly inhibit Rb, offering a predisposition to tumorigenesis, but other factors are likely needed for lymphomagenesis.

LANA is also known to activate the Wnt and Notch signaling pathways. It interacts with glycogen synthetase kinase 3β (GSK-3β), which typically phosphorylates and inactivates β-catenin through ubiquitin-mediated proteolysis [61]. However, upon LANA binding of GSK-3β, GSK-3β is relocated from the cytosol to the nucleus, which allows for the accumulation of β-catenin. The dysregulated β-catenin can then oligomerize the transcription factor LEF for the targeted transcription of Wnt genes, such as cyclin D, c-Myc, and c-Jun, which promote cell proliferation and survival. PEL cells show an upregulation of β-catenin [62]. The LANA-mediated alteration in the Wnt/β-catenin pathway further implicates LANA as a viral oncogene. LANA modulates Notch signaling, and the dysregulation of Notch signaling is implicated in its contribution to oncogenesis because Notch is important in the transcriptional activation of genes related to cell cycle, differentiation, and proliferation. The stability of Notch is negatively regulated by the E3 ligase Sel10. LANA has been shown to bind Sel10, suppressing the degradation of Notch, thus enhancing KSHV-infected cell proliferation [63]. Another function of LANA is upregulating telomerase activity by its interaction with transcription factor Sp1, contributing to cellular immortalization by maintaining telomere length [64].

LANA evades the host immune response by downregulating the expression of major histocompatibility complex (MHC) class I molecules [65], allowing infected cells to evade recognition and destruction from cytotoxic T cells. Epigenetically, LANA manipulates the cell through histone modifications and altering DNA methylation patterns. By interacting with histone-modifying enzymes, LANA alters chromatin structure, critical to regulating the expression of viral and host genes. During de novo infection, LANA recruits DNA methyltransferase 3A (DNMT3A) to broadly silence gene activity of the KSHV genome, allowing it to limit viral gene expression [66]. In summary, LANA interacts with a variety of cellular proteins to modulate their function and create a cell environment favoring viral latency and oncogenesis.

### 4.3. Viral Homologs Mimic Cellular Counterparts, Promoting Lymphomagenesis

Similar to other herpesviruses, KSHV has co-evolved with its human host and adapted a strategy of molecular mimicry by encoding a number of viral genes, such as *vCyclin*, *vFLIP*, *vGPCR*, and *vIRFs*, with homology to human genes (Table 1) [13,67].

In this review, we chose to focus on vIL-6, vCyclin, vFLIP, and vIRF3 because they are viral mimics that are well studied in the context of KSHV-associated lymphomagenesis. We also describe the viral microRNAs.

#### 4.3.1. Viral Interleukin-6 (vIL-6)

KSHV encodes vIL-6, a viral homolog of human IL-6 (hIL-6), which has optimized IL-6 functions (reviewed in [37]). vIL-6 shares 25% sequence identity and 63% amino acid similarity with hIL-6; however, it can stimulate all known hIL-6 pathways [68]. vIL-6 differs from hIL-6 in several ways. vIL-6 can signal by engaging merely the gp130 subunit of the IL-6 receptor, whereas hIL-6 requires the full-length IL-6 receptor with both gp130 and gp80. vIL-6 also has broader capabilities in a wider variety of cell types compared to hIL-6 due to its ability to engage the ubiquitously expressed gp130 alone. Relative to hIL-6, vIL-6 secretion is considered inefficient because most of the protein is retained within the endoplasmic reticulum (ER). vIL-6 is expressed during both latency and lytic replication but to a much higher degree during the lytic phase. vIL-6 is detectable in sera and/or tissue samples from all three KSHV-associated malignancies [69].

Within the ER, the ER transmembrane protein vitamin K epoxide reductase complex subunit 1 variant 2 (VKORC1v2) is a binding partner of vIL-6, and the interaction contributes to growth and anti-apoptosis of PEL cells [70]. Additionally, vIL-6 has been shown to promote productive replication and latent PEL cell viability by upregulating the mannose-6-phosphate- and peptide hormone-interacting receptor IGF2R by preventing IGF2R binding with VKORC1cv2, rescuing IGF2R from degradation [71]. The rescued IGF2R, in turn, promotes PEL cell viability.

The role of vIL-6 in tumor development has been evaluated in several studies. vIL-6 has been shown to transform murine fibroblast NIH 3T3 cells, and when mice were inoculated with vIL-6-producing clones, the injected vIL-6-positive cells formed larger tumors relative to control cells [72]. Another group generated a vIL-6 transgenic mouse model using the BALB/c (C) genetic background because of its hyper-susceptibility to malignant plasma cell tumors. The C.vIL6 mice were prone to severe and sometimes fatal MCD-like disease [35]. The C.vIL6iMyc mice with transgenes for vIL6 and a chromosomal translocation to deregulate *Myc* in B cells developed aggressive plasmablastic neoplasms with both clinical and histopathological similarities to PEL, where immunodeficient RAG2^−/−^ mice were transplanted with the BJAB B cell line containing either WT KSHV or a mutant strain without vIL-6 [36]. While the vIL-6 gene did not affect tumor mass, when vIL-6 was present, significantly more tumors were detected [73]. This indicates the critical role of vIL-6 in KSHV tumor formation in B cells in vivo.

vIL-6 contributes to lymphomagenesis through autocrine and paracrine signaling, activation of the JAK/STAT pathway, angiogenesis, inflammation, immune modulation, synergistic effects with other viral proteins, and direct effects on B cells. vIL-6 acts in both an autocrine and paracrine manner, where it can act on the cell producing vIL-6 as well as neighboring cells (whether infected or not) to create a pro-inflammatory and tumorigenic microenvironment by promoting the proliferation and survival of cells. Importantly, vIL-6 activates STAT3 through the JAK/STAT pathway [37]. Activated STAT3 translocates to the nucleus and promotes the transcription of genes involved in cell proliferation, survival, and immune evasion, including Bcl-2, Bcl-xL, cyclin D1, and c-Myc. vIL-6 signaling both phosphorylates and acetylates STAT3. Phosphorylated STAT3 transcriptionally activates DNA methyltransferase 1 (DNMT1) expression. Acetylated STAT3 forms a transcription factor complex with DNMT1 to bind and methylate the *CAV1* promoter for *CAV1* silencing. Downregulated CAV1 leads to activation of Akt signaling for increased cell invasion and growth transformation induced by KSHV [74]. Additionally, vIL-6 induces the production of a key angiogenesis factor, vascular endothelial growth factor (VEGF) [72]. Enhanced angiogenesis supplies the growing tumor with oxygen and nutrients, contributing to tumor growth and metastasis.

Recently, vIL-6 has been shown to promote the SIRT3-induced deacetylation of SERBP1 [75]. This prevents SERBP1 binding to and the protection of lipoyltransferase 2 mRNA from degradation, thus preventing ferroptosis. Ferroptosis is a form of cell death characterized by iron accumulation and lipid peroxidation that is known to restrict pathogenic infections and act as an innate tumor suppressor mechanism. Through SIRT3 and SERBP1, vIL-6 inhibits ferroptosis, thus allowing for KSHV-induced cell transformation.

Given that KSHV-associated PEL and MCD are often observed in the context of HIV patients, it has been proposed that HIV-1 and KSHV may synergize to promote angiogenesis and tumorigenesis. Studies show that both HIV-1 trans-activator of transcription (Tat) and negative factor (Nef) synergize with KSHV vIL-6 to promote vascular tube formation and cell proliferation, enhancing oncogenesis by activating the AKT pathway [76,77].

#### 4.3.2. Viral Cyclin (vCyclin)

**Figure 2:**
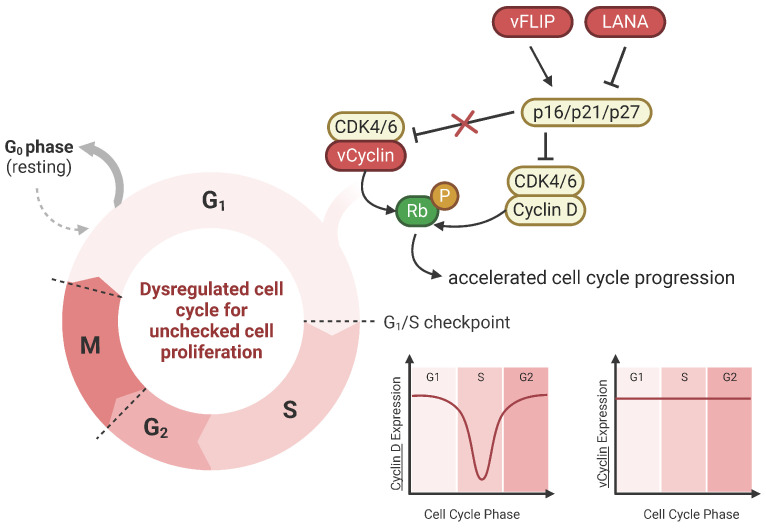
vCyclin dysregulates cell cycle for uncontrolled cell proliferation. vCyclin is the viral homolog of cellular cyclin D. vCyclin complexes with CDK6 and phosphorylates tumor suppressor protein Rb, bypassing the G1/S checkpoint. Cellular tumor suppressor proteins p16, p21, and p27 are cyclin-dependent kinase inhibitors that block cyclin D. However, unlike cyclin D, vCyclin is resistant to these CDK inhibitors, which contributes to cell cycle dysregulation. Additionally, other KSHV viral proteins like vFLIP and LANA regulate these cellular CDK inhibitors. Cyclin D expression is cell-cycle-phase-dependent; however, vCyclin expression remains stabilized throughout, further allowing for accelerated cell cycle progression and unchecked cell proliferation.

vCyclin mimics cellular cyclin D and E and activates cyclin-dependent kinases (CDK), namely CDK6, to drive cell cycle progression from the G1 to S phase (Figure 2) [78,79]. Human cyclin D1 is an important regulator of the cell cycle, where in normal cells, cyclin D1 expression levels are strictly regulated. However, when cyclin D1 expression is high, cyclin D1 drives unchecked cell proliferation, promoting tumor growth, and it is central to the pathogenesis of cancer. Like the cyclin D1-CDK6 complex, the vCyclin-CDK6 complex can phosphorylate and inactivate Rb, a critical regulator of the G1/S checkpoint [79]. Rb inactivation allows the cell to override cell cycle checkpoints and enter the S phase, leading to uncontrolled cell proliferation. Distinct from its cellular counterpart, the vCyclin-CDK6 complex is resistant to inhibition by cyclin-dependent kinase inhibitors like p21 and p27 [80]. Insensitivity allows for continued cell cycle progression, even though these inhibitory signals would typically halt cell division. Furthermore, while cyclin D expression is cell-cycle-dependent, vCyclin levels remain stable regardless of the cell cycle phase. The extended expression of vCyclin could contribute to the constitutive activation of vCyclin-CDK complexes in KSHV-infected cells [81,82].

vCyclin synergizes with other viral oncogenes, LANA and vFLIP. vCyclin and vFLIP are co-expressed from a bicistronic mRNA and are functionally interdependent on one another. vFLIP expression results in p21-/p27-mediated G1 arrest due to NF-kB hyperactivation; however, vCyclin prevents G1 arrest, promoting cell cycle entry [83]. Thus, vCyclin and vFLIP cooperate with one another. A study in lymphatic endothelial cells demonstrated that vCyclin is essential for bypassing senescence and thus may play a role in KS tumor development [84]; however, further studies are needed to show if this applies to B cells for PEL and MCD tumorigenesis.

vCyclin can also alter cell metabolism. Insufficient oxygen levels in tissues, or hypoxia, induces pathways that promote tumor growth. Several studies show that hypoxia plays an important role in KSHV-induced tumors, where KS and PEL preferentially develop in locations with hypoxia [85]. Hypoxia is mediated by hypoxia-inducible factors (HIFs). Typically, the cell response to hypoxia with high HIF-1α levels induces metabolic reprogramming that restricts DNA replication and arrests cell division; however, KSHV-infected cells can maintain replication in hypoxia. A study showed that vCyclin can modulate HIF-1α signaling by binding HIF-1α for eventual lysosomal degradation through cytosolic translocation to restrict HIF-1α transcriptional activity [86]. Thus, vCyclin overcomes HIF-1α-mediated replication arrest and induces an oncogenic phenotype. A recent study found another way that KSHV alters metabolic pathways by hijacking nucleotide biosynthesis and deamidation-mediated glycolysis [87]. vCyclin binds CDK6 to phosphorylate carbamoyl-phosphate synthetase 2, aspartate transcarbamoylase, and dihydroorotase (CAD), a key enzyme of pyrimidine synthesis that deamidates the NF-kB subunit RelA to promote aerobic glycolysis and cell proliferation. KSHV-mediated metabolic reprogramming through vCyclin contributes to PEL tumorigenesis, shown in vitro and in vivo.

#### 4.3.3. Viral FLICE Inhibitory Protein (vFLIP)

vFLIP is encoded from the same major latency locus as LANA and vCyclin and is the viral homolog of cellular FLICE inhibitory protein (FLIP). vFLIP is expressed during latency and undergoes RTA-induced degradation during lytic reactivation. Cellular FLIPs bind adaptor proteins in the Fas pathway through their death effector domains [88]. This binding impairs the recruitment and activation of caspase 8, blocking the caspase activation cascade, resulting in programmed cell death. Thus, vFLIP inhibits caspase-8, a key initiator of the extrinsic apoptosis pathway, allowing for survival and proliferation of infected cells (reviewed in [89]).

vFLIP also blocks cell death by activating NF-kB signaling [88,90,91]. vFLIP complexes with IKKγ, activating IKK, which leads to the phosphorylation of IkB and the release of active NF-kB. PEL cells display high levels of NF-kB activity [39]. Additionally, vFLIP promotes NF-kB signaling by upregulating IKKε and CADM1 and via the inhibition of the SAP18/HDAC1 complex, leading to NF-kB activation via the acetylation of p65 [92]. Thus, vFLIP is important in PEL cells to prevent cell death and upregulate NF-kB. NF-kB signaling leads to the transcription of cell proliferation and survival genes. Chronic activation of NF-kB augments the inflammatory tumor microenvironment that contributes to the progression of KSHV-associated lymphoproliferation. In a transgenic model of vFLIP, there was persistent NF-kB activation and an increased incidence of lymphomas as well as B cell abnormalities, consistent with those seen in MCD [90]. Another study showed that vFLIP increases LINE-1 retrotransposition, promoting genomic instability, a hallmark of cancer [93].

Hyperinflammation is a hallmark of KS-associated malignancies. A screen for inhibitors of KSHV-induced oncogenesis identified several anti-inflammatory candidates, including dexamethasone, which activates glucocorticoid receptor signaling. The group found that in KSHV-transformed cells, particularly, interleukin-1 alpha (IL-1α) was most induced, while IL-1 receptor antagonist (IL-1Ra) was highly suppressed [94]. KSHV micro RNAs and vFLIP were found to suppress IL-1Ra. Furthermore, vFLIP and vCyclin were found to inhibit glucocorticoid signaling in KSHV-transformed cells.

#### 4.3.4. Viral Interferon Regulatory Factor 3 (vIRF3)

KSHV encodes multiple viral interferon regulatory factors (IRF) that interfere with the host interferon response. The KSHV genome has at least four genes encoding IRFs [95]. Cellular IRFs are transcription factors that promote the activation of type I interferons. However, vIRFs inhibit type I interferon signaling, which allows KSHV to evade a host’s antiviral immune response. Thus, vIRFs promote cell survival and proliferation by modulating immune and inflammatory pathways.

While most of the vIRFs are lytic proteins, vIRF3, also known as LANA-2, encodes a nuclear protein expressed in latency [18]. However, latent vIRF3 gene expression differs by cell type. vIRF3 is expressed in latently infected B cells, including both PEL cells and cells from MCD but not KS spindle cells [96]. This implicates vIRF3 in the progression of KSHV-associated lymphomagenesis. vIRF3 is known to interact with p53 and reduce its stability, preventing its DNA binding activity [97]. Thus, the pro-apoptotic function of p53 is blocked, prolonging the lifespan of KSHV-infected B cells. Additionally, vIRF3 stimulates transcriptional activity of the oncogene Myc. vIRF3 competes with c-Myc to bind Myc modulator 1 (MM-1α), which reverses the MM-1α suppression of c-Myc. Furthermore, vIRF3 binds to the F-box of Skp2, part of a ubiquitin ligase complex, to recruit it to c-Myc-regulated promotors for the activation of c-Myc dependent transcription [98].

#### 4.3.5. Viral microRNAs (miRNA)

miRNAs are non-coding single-stranded RNAs that are approximately 20–24 nucleotides long. They primarily function post-transcriptionally to inhibit target gene expression by binding to the complementary sequence of the target gene to compromise its expression by mRNA degradation or inhibition of translation. KSHV encodes 12 distinct pre-miRNAs that are later processed into 25 mature miRNAs in its latency-associated region of the viral genome [99,100,101]. KSHV-infected cells can release exosomes carrying viral miRNAs, affecting neighboring B cells in a paracrine manner, contributing to KSHV pathogenesis in PEL and MCD [102]. KSHV miRNAs are expressed at different levels, and the miRNA expression level also differs among PEL cell lines, where some PEL lines express low levels (BC-1, BCBL-1) while others express high levels (BC-3) [103]. The expression level of miRNAs correlates to the impact of their function [104]. A study using PAR-CLIP allowed for the transcriptome-wide identification of over 2000 cellular targets of KSHV miRNAs in PEL cells (BC-1, BCBL1, and BC-3), implicating KSHV miRNAs in virus infection latency and lymphomagenesis [103].

Several KSHV miRNAs are expressed in latency and support latency maintenance. miR-K9-5p targets the KSHV replication and transcription activator (RTA), which is critical for initiating KSHV lytic reactivation [105]. Both miR-K3 and miR-K1 stabilize KSHV latency by regulating the RTA promoter, where miR-K3 targets nuclear factor I/B and miR-K1 regulates NF-κB signaling by targeting IκBα [106,107]. miR-K11 also stabilizes KSHV latency by attenuating the interferon response induced by pattern recognition receptors [108]. A study using CRISPR/Cas9 to ablate KSHV miRNA expression in PEL cell lines, BCBL-1 and BCP-1, led to the upregulation of KSHV lytic genes, further demonstrating the significance of KSHV miRNAs in maintaining latency [109].

There are KSHV miRNAs that share seed homology to cellular miRNAs, suggesting that these viral miRNAs mimic their human counterpart. miR-K11 has sequence similarity to human miR-155, which plays a role in B cell development and oncogenesis [110,111]. miR-K11 has been shown to target *BACH1* mRNA for increased viability under oxidative stress and the enhanced proliferation of CD19^+^ B cells in the spleen [110,111,112]. Thus, it is thought that miR-K11 has a role in KSHV-induced lymphomagenesis. miR-K10a and cellular miR-142-3p are also analogs and share some seed homology. miR-142-3p is known to have high abundance in PEL cells. miR-K10a has been shown to overlap in function with miR-142-3p, and it represses cell cycle inhibitor p27 in murine fibroblasts and PEL cells; thus, it has transforming activity [24]. Additionally, miR-K10 and its variants inhibit TGF-β-regulated cell growth and apoptosis in PEL cells, contributing to the dysregulation of normal cell growth control [113]. Other KSHV miRNAs that inhibit cell death include miR- K1, K3, and K4. These miRNAs target *CASP3* to block caspase-induced apoptosis [114]. miR-K1 also targets p21, an inhibitory regulator of the cell cycle; thus, it blocks cell cycle arrest [115]. KSHV miRNAs maintain latency and dysregulate cell growth and death, promoting lymphomagenesis.

## 5. Therapeutics

**Figure 3:**
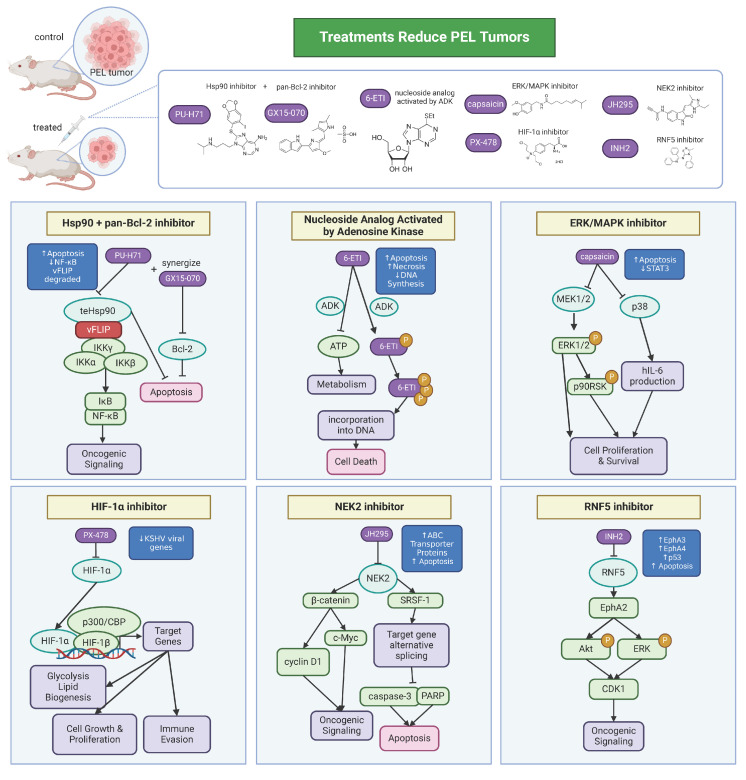
Therapeutics for PEL. KSHV co-opts host factors and machinery to promote its pathogenesis and persistence, dysregulating normal cell processes that then lead to oncogenic signaling. Development of therapeutics targeted towards host factors has shown efficacy in treating PEL-associated cell lines and mouse tumor models. Many of the therapeutics work to increase tumor cell death via apoptosis.

Based on studies of KSHV, PEL, and MCD, there has been progress in the development of therapeutics. PEL is highly aggressive and frequently resistant to standard chemotherapy regimens like CHOP (cyclophosphamide, doxorubicin, vincristine, and prednisone). Thus, therapeutics have been developed that target cellular products (Figure 3).

Hsp90 is a chaperone protein that has been shown to be essential in the survival of KSHV-associated malignancies. vFLIP forms a complex with the host proteins IKKγ and Hsp90. Studies have shown that inhibiting Hsp90 blocked viral gene expression in primary infection and downregulated NF-κB activity [91,116]. Another study showed that treatment with a purine scaffold Hsp90 inhibitor, PU−H71, selectively binds the fraction of Hsp90 that is enriched in tumor cells. PU−H71 kills PEL cells and prolonged disease-free survival in a PEL xenograft mouse model [117]. Furthermore, PU−H71 and a pan-Bcl2 inhibitor, GX15−070, synergize in PEL cells to enhance PEL cell killing [117]. In a follow-up screen to identify additional PEL-dependencies, the nucleoside analog activated by adenosine kinase, 6−ETI, was a top hit. 6−ETI was validated to be effective in reducing tumors in a PEL xenograft model by inducing S phase arrest [118]. It has also been shown that PEL relies on the mitotic kinase NEK2 for survival [119]. Inhibition of NEK2 with the JH295 inhibitor resulted in the caspase-3 mediated apoptosis of PEL and prolonged survival and reduced the tumor burden in a PEL mouse model. JH295 also sensitizes lymphomas to other chemotherapeutic agents like rapamycin, enhancing cancer cell death [120,121]. Another group found that the inhibition of Ring finger protein 5 (RNF5), an ER-localized E3 ligase, decreases PEL xenograft tumor growth and downregulates both viral and cell cycle gene expression [122]. The RNF5 inhibitor INH2 works by increasing the levels of Ephrin receptors A3 and A4, reducing Erk and Akt activation as well as oncogenic KSHV lytic replication. Interestingly, a group found that capsaicin, a compound from chili pepper, can inhibit the growth of PEL cells by inhibiting ERK, p38 MAPK, and hIL-6 expression [123]. By inhibiting ERK and p38 MAPK phosphorylation, capsaicin can induce apoptosis in PEL cells in a caspase-9-dependent manner. This was confirmed in vitro in PEL cells and in PEL development in SCID mice. HIF-1α has been considered as a therapeutic target for PEL. HIF-1α is necessary for creating a metabolic state optimal for PEL growth, where HIF-1α knockdown or treatment with PX−478, a small-molecule inhibitor of HIF-1α, leads to a decreased expression of KSHV latent genes [124]. Thus, inhibition of HIF-1α could be a potential therapeutic strategy.

For KSHV-MCD, the monoclonal antibody drug rituximab has been used in patients, improving the survival of these patients and reducing the risk of lymphoma [125]. Additionally, a combination therapy with rituximab and the anthracycline doxorubicin has been used with efficacy in patients with MCD and KS [126]. Monoclonal antibodies targeting the IL-6/IL-6 receptor have been approved for idiopathic MCD but are not treatment strategies for KSHV-MCD [127]. Given the role of vIL-6 in both MCD and PEL, there is compelling evidence supporting the utility of vIL-6 neutralization, and there are ongoing efforts to generate vIL-6-specific neutralizing antibodies [68].

Generally, for KSHV-associated malignancies, viral and host products contributing to KSHV lymphomagenesis are potential therapeutic targets. For example, therapies have been developed to inhibit viral oncogenes, like LANA [128], or to inhibit the signaling pathways involved, like the JAK/STAT pathway. JAK inhibitors have been studied to block the pathogenesis of both PEL and KSHV-MCD, given the importance of the IL-6/JAK/STAT3 axis in both diseases. From a panel of JAK inhibitors, pacritinib was found to most significantly inhibit the growth of PEL cell lines [129].

## 6. Conclusions

KSHV is the driver of the lymphoproliferative disorders PEL and MCD. KSHV encodes genes to commandeer cellular processes to benefit KSHV persistence, which gives way to lymphoproliferation through a dysregulated cell cycle and immune evasion. Furthering our understanding of the molecular mechanisms of KSHV-associated lymphomagenesis informs the development of more effective treatments. Active areas of investigation include studying the interactions between KSHV proteins and host cellular pathways, looking into ways to enhance the immune response against KSHV-infected cells, and developing drugs targeted to KSHV-induced epigenetic modifications.

## Figures and Tables

**Table 1:** KSHV viral genes with human gene homologs.

Human Gene	Viral Gene	KSHV ORF	Viral Function
*Bcl-2* *CyclinD* *DUB*	*vBcl-2* *vCyclin* *ORF64*	ORF16 ORF72 ORF64	Pro-apoptotic factor signaling through caspases Complexes with CDK6 to drive G1 to S phase cell cycle transition Viral deubiquitinase
*FLIP*	*vFLIP*	ORF71	Activates NF-κB signaling
*GPCR*	*vGPCR*	ORF74	Constitutively activates G-protein-coupled receptor for angiogenesis
*IL-6*	*vIL-6*	ORF K2	Cytokine that activates the JAK/STAT pathway
*IRF4/IRF8* *MARCH ligases* *MIP1α* *OX2*	*vIRF3* *vIRF1* *vIRF2* *vIRF4* *MIR1* *MIR2* *vMIP-I* *vMIP-II* *vMIP-III* *vOX2*	ORF K10.5 and K10.6 ORF K9 ORF K11 and K11.1 ORF K10 ORF K3 ORF K5 ORF K6 ORF K4 ORF K4.1 ORF K14	The vIRFs are transcription factors that bind cellular IRFs and inhibit interferon signaling, p53, and apoptosis Ubiquitin E3 ligases that target MHC class I molecules for degradation Binds CCR8 for Ca^2+^-ion-dependent signaling Agonist of CCR3 and CCR8 and antagonist of chemokine receptors Binds CCR4 as chemoattractant of Th2 T cells Modulates inflammatory responses

## References

[1] Cesarman E., Damania B., Krown S.E., Martin J., Bower M., Whitby D. (2019). Kaposi sarcoma. Nat. Rev. Dis. Primers.

[2] Chang Y., Cesarman E., Pessin M.S., Lee F., Culpepper J., Knowles D.M., Moore P.S. (1994). Identification of herpesvirus-like DNA sequences in AIDS-associated Kaposi’s sarcoma. Science.

[3] Soulier J., Grollet L., Oksenhendler E., Cacoub P., Cazals-Hatem D., Babinet P., d’Agay M.F., Clauvel J.P., Raphael M., Degos L. (1995). Kaposi’s sarcoma-associated herpesvirus-like DNA sequences in multicentric Castleman’s disease. Blood.

[4] Cesarman E., Chang Y., Moore P.S., Said J.W., Knowles D.M. (1995). Kaposi’s sarcoma-associated herpesvirus-like DNA sequences in AIDS-related body-cavity-based lymphomas. N. Engl. J. Med..

[5] Broussard G., Damania B. (2020). Regulation of KSHV Latency and Lytic Reactivation. Viruses.

[6] Manners O., Murphy J.C., Coleman A., Hughes D.J., Whitehouse A. (2018). Contribution of the KSHV and EBV lytic cycles to tumourigenesis. Curr. Opin. Virol..

[7] Chakraborty S., Veettil M.V., Chandran B. (2012). Kaposi’s Sarcoma Associated Herpesvirus Entry into Target Cells. Front. Microbiol..

[8] Thandra K.C., Barsouk A., Saginala K., Padala S.A., Barsouk A., Rawla P. (2021). Epidemiology of Non-Hodgkin’s Lymphoma. Med. Sci..

[9] Morton L.M., Wang S.S., Cozen W., Linet M.S., Chatterjee N., Davis S., Severson R.K., Colt J.S., Vasef M.A., Rothman N. (2008). Etiologic heterogeneity among non-Hodgkin lymphoma subtypes. Blood.

[10] https://www.lls.org/lymphoma/non-hodgkin-lymphoma/nhl-subtypes.

[11] Jaffe E.S., Harris N.L., Stein H., Isaacson P.G. (2008). Classification of lymphoid neoplasms: The microscope as a tool for disease discovery. Blood.

[12] Chu Y., Liu Y., Fang X., Jiang Y., Ding M., Ge X., Yuan D., Lu K., Li P., Li Y. (2023). The epidemiological patterns of non-Hodgkin lymphoma: Global estimates of disease burden, risk factors, and temporal trends. Front. Oncol..

[13] Giffin L., Damania B. (2014). KSHV: Pathways to tumorigenesis and persistent infection. Adv. Virus Res..

[14] Nador R.G., Cesarman E., Chadburn A., Dawson D.B., Ansari M.Q., Sald J., Knowles D.M. (1996). Primary effusion lymphoma: A distinct clinicopathologic entity associated with the Kaposi’s sarcoma-associated herpes virus. Blood.

[15] Jones K.D., Aoki Y., Chang Y., Moore P.S., Yarchoan R., Tosato G. (1999). Involvement of interleukin-10 (IL-10) and viral IL-6 in the spontaneous growth of Kaposi’s sarcoma herpesvirus-associated infected primary effusion lymphoma cells. Blood.

[16] Lurain K., Polizzotto M.N., Aleman K., Bhutani M., Wyvill K.M., Goncalves P.H., Ramaswami R., Marshall V.A., Miley W., Steinberg S.M. (2019). Viral, immunologic, and clinical features of primary effusion lymphoma. Blood.

[17] Godfrey A., Anderson J., Papanastasiou A., Takeuchi Y., Boshoff C. (2005). Inhibiting primary effusion lymphoma by lentiviral vectors encoding short hairpin RNA. Blood.

[18] Wies E., Mori Y., Hahn A., Kremmer E., Sturzl M., Fleckenstein B., Neipel F. (2008). The viral interferon-regulatory factor-3 is required for the survival of KSHV-infected primary effusion lymphoma cells. Blood.

[19] Horenstein M.G., Nador R.G., Chadburn A., Hyjek E.M., Inghirami G., Knowles D.M., Cesarman E. (1997). Epstein-Barr virus latent gene expression in primary effusion lymphomas containing Kaposi’s sarcoma-associated herpesvirus/human herpesvirus-8. Blood.

[20] Szekely L., Chen F., Teramoto N., Ehlin-Henriksson B., Pokrovskaja K., Szeles A., Manneborg-Sandlund A., Lowbeer M., Lennette E.T., Klein G. (1998). Restricted expression of Epstein-Barr virus (EBV)-encoded, growth transformation-associated antigens in an EBV- and human herpesvirus type 8-carrying body cavity lymphoma line. J. Gen. Virol..

[21] Lange P.T., Damania B. (2020). Modeling oncogenic herpesvirus infections in humanized mice. Curr. Opin. Virol..

[22] Bigi R., Landis J.T., An H., Caro-Vegas C., Raab-Traub N., Dittmer D.P. (2018). Epstein-Barr virus enhances genome maintenance of Kaposi sarcoma-associated herpesvirus. Proc. Natl. Acad. Sci. USA.

[23] McHugh D., Caduff N., Barros M.H.M., Ramer P.C., Raykova A., Murer A., Landtwing V., Quast I., Styles C.T., Spohn M. (2017). Persistent KSHV Infection Increases EBV-Associated Tumor Formation In Vivo via Enhanced EBV Lytic Gene Expression. Cell Host Microbe.

[24] Munz C. (2021). The Role of Lytic Infection for Lymphomagenesis of Human gamma-Herpesviruses. Front. Cell. Infect. Microbiol..

[25] Faure A., Hayes M., Sugden B. (2019). How Kaposi’s sarcoma-associated herpesvirus stably transforms peripheral B cells towards lymphomagenesis. Proc. Natl. Acad. Sci. USA.

[26] Fajgenbaum D.C., van Rhee F., Nabel C.S. (2014). HHV-8-negative, idiopathic multicentric Castleman disease: Novel insights into biology, pathogenesis, and therapy. Blood.

[27] Zhou T., Wang H.W., Pittaluga S., Jaffe E.S. (2021). Multicentric Castleman disease and the evolution of the concept. Pathologica.

[28] Ramaswami R., Lurain K., Polizzotto M.N., Ekwede I., Waldon K., Steinberg S.M., Mangusan R., Widell A., Rupert A., George J. (2021). Characteristics and outcomes of KSHV-associated multicentric Castleman disease with or without other KSHV diseases. Blood Adv..

[29] Oksenhendler E., Boutboul D., Fajgenbaum D., Mirouse A., Fieschi C., Malphettes M., Vercellino L., Meignin V., Gerard L., Galicier L. (2018). The full spectrum of Castleman disease: 273 patients studied over 20 years. Br. J. Haematol..

[30] Vega F., Miranda R.N., Medeiros L.J. (2020). KSHV/HHV8-positive large B-cell lymphomas and associated diseases: A heterogeneous group of lymphoproliferative processes with significant clinicopathological overlap. Mod. Pathol..

[31] Dossier A., Meignin V., Fieschi C., Boutboul D., Oksenhendler E., Galicier L. (2013). Human herpesvirus 8-related Castleman disease in the absence of HIV infection. Clin. Infect. Dis..

[32] Aoki Y., Tosato G., Fonville T.W., Pittaluga S. (2001). Serum viral interleukin-6 in AIDS-related multicentric Castleman disease. Blood.

[33] Aoki Y., Jones K.D., Tosato G. (2000). Kaposi’s sarcoma-associated herpesvirus-encoded interleukin-6. J. Hematother. Stem Cell Res..

[34] Chadburn A., Hyjek E.M., Tam W., Liu Y., Rengifo T., Cesarman E., Knowles D.M. (2008). Immunophenotypic analysis of the Kaposi sarcoma herpesvirus (KSHV; HHV-8)-infected B cells in HIV+ multicentric Castleman disease (MCD). Histopathology.

[35] Suthaus J., Stuhlmann-Laeisz C., Tompkins V.S., Rosean T.R., Klapper W., Tosato G., Janz S., Scheller J., Rose-John S. (2012). HHV-8-encoded viral IL-6 collaborates with mouse IL-6 in the development of multicentric Castleman disease in mice. Blood.

[36] Rosean T.R., Holman C.J., Tompkins V.S., Jing X., Krasowski M.D., Rose-John S., Janz S. (2016). KSHV-encoded vIL-6 collaborates with deregulated c-Myc to drive plasmablastic neoplasms in mice. Blood Cancer J..

[37] Sakakibara S., Tosato G. (2011). Viral interleukin-6: Role in Kaposi’s sarcoma-associated herpesvirus: Associated malignancies. J. Interferon Cytokine Res..

[38] Shaffer A.L., Young R.M., Staudt L.M. (2012). Pathogenesis of human B cell lymphomas. Annu. Rev. Immunol..

[39] Keller S.A., Hernandez-Hopkins D., Vider J., Ponomarev V., Hyjek E., Schattner E.J., Cesarman E. (2006). NF-kappaB is essential for the progression of KSHV- and EBV-infected lymphomas in vivo. Blood.

[40] Bhatt A.P., Damania B. (2012). AKTivation of PI3K/AKT/mTOR signaling pathway by KSHV. Front. Immunol..

[41] Xiang Q., Yang Z., Nicholas J. (2022). STAT and Janus kinase targeting by human herpesvirus 8 interferon regulatory factor in the suppression of type-I interferon signaling. PLoS Pathog..

[42] Friborg J., Kong W., Hottiger M.O., Nabel G.J. (1999). p53 inhibition by the LANA protein of KSHV protects against cell death. Nature.

[43] Hume A.J., Kalejta R.F. (2009). Regulation of the retinoblastoma proteins by the human herpesviruses. Cell Div..

[44] Bhatt A.P., Jacobs S.R., Freemerman A.J., Makowski L., Rathmell J.C., Dittmer D.P., Damania B. (2012). Dysregulation of fatty acid synthesis and glycolysis in non-Hodgkin lymphoma. Proc. Natl. Acad. Sci. USA.

[45] Gregory S.M., Davis B.K., West J.A., Taxman D.J., Matsuzawa S., Reed J.C., Ting J.P., Damania B. (2011). Discovery of a viral NLR homolog that inhibits the inflammasome. Science.

[46] West J., Damania B. (2008). Upregulation of the TLR3 pathway by Kaposi’s sarcoma-associated herpesvirus during primary infection. J. Virol..

[47] Lagos D., Vart R.J., Gratrix F., Westrop S.J., Emuss V., Wong P.P., Robey R., Imami N., Bower M., Gotch F. (2008). Toll-like receptor 4 mediates innate immunity to Kaposi sarcoma herpesvirus. Cell Host Microbe.

[48] West J.A., Wicks M., Gregory S.M., Chugh P., Jacobs S.R., Zhang Z., Host K.M., Dittmer D.P., Damania B. (2014). An important role for mitochondrial antiviral signaling protein in the Kaposi’s sarcoma-associated herpesvirus life cycle. J. Virol..

[49] Kerur N., Veettil M.V., Sharma-Walia N., Bottero V., Sadagopan S., Otageri P., Chandran B. (2011). IFI16 acts as a nuclear pathogen sensor to induce the inflammasome in response to Kaposi Sarcoma-associated herpesvirus infection. Cell Host Microbe.

[50] Ma Z., Jacobs S.R., West J.A., Stopford C., Zhang Z., Davis Z., Barber G.N., Glaunsinger B.A., Dittmer D.P., Damania B. (2015). Modulation of the cGAS-STING DNA sensing pathway by gammaherpesviruses. Proc. Natl. Acad. Sci. USA.

[51] Wu J.J., Li W., Shao Y., Avey D., Fu B., Gillen J., Hand T., Ma S., Liu X., Miley W. (2015). Inhibition of cGAS DNA Sensing by a Herpesvirus Virion Protein. Cell Host Microbe.

[52] Inn K.S., Lee S.H., Rathbun J.Y., Wong L.Y., Toth Z., Machida K., Ou J.H., Jung J.U. (2011). Inhibition of RIG-I-mediated signaling by Kaposi’s sarcoma-associated herpesvirus-encoded deubiquitinase ORF64. J. Virol..

[53] Zhang H., Ni G., Damania B. (2020). ADAR1 Facilitates KSHV Lytic Reactivation by Modulating the RLR-Dependent Signaling Pathway. Cell Rep..

[54] Zhang G., Chan B., Samarina N., Abere B., Weidner-Glunde M., Buch A., Pich A., Brinkmann M.M., Schulz T.F. (2016). Cytoplasmic isoforms of Kaposi sarcoma herpesvirus LANA recruit and antagonize the innate immune DNA sensor cGAS. Proc. Natl. Acad. Sci. USA.

[55] Broussard G., Ni G., Zhang Z., Li Q., Cano P., Dittmer D.P., Damania B. (2023). Barrier-to-autointegration factor 1 promotes gammaherpesvirus reactivation from latency. Nat. Commun..

[56] Uppal T., Banerjee S., Sun Z., Verma S.C., Robertson E.S. (2014). KSHV LANA—The master regulator of KSHV latency. Viruses.

[57] Cotter M.A., Robertson E.S. (1999). The latency-associated nuclear antigen tethers the Kaposi’s sarcoma-associated herpesvirus genome to host chromosomes in body cavity-based lymphoma cells. Virology.

[58] Ye X., Guerin L.N., Chen Z., Rajendren S., Dunker W., Zhao Y., Zhang R., Hodges E., Karijolich J. (2024). Enhancer-promoter activation by the Kaposi sarcoma-associated herpesvirus episome maintenance protein LANA. Cell Rep..

[59] Chen W., Hilton I.B., Staudt M.R., Burd C.E., Dittmer D.P. (2010). Distinct p53, p53:LANA, and LANA complexes in Kaposi’s Sarcoma--associated Herpesvirus Lymphomas. J. Virol..

[60] Platt G., Carbone A., Mittnacht S. (2002). p16INK4a loss and sensitivity in KSHV associated primary effusion lymphoma. Oncogene.

[61] Fujimuro M., Hayward S.D. (2003). The latency-associated nuclear antigen of Kaposi’s sarcoma-associated herpesvirus manipulates the activity of glycogen synthase kinase-3beta. J. Virol..

[62] Fujimuro M., Wu F.Y., ApRhys C., Kajumbula H., Young D.B., Hayward G.S., Hayward S.D. (2003). A novel viral mechanism for dysregulation of beta-catenin in Kaposi’s sarcoma-associated herpesvirus latency. Nat. Med..

[63] Lan K., Verma S.C., Murakami M., Bajaj B., Kaul R., Robertson E.S. (2007). Kaposi’s sarcoma herpesvirus-encoded latency-associated nuclear antigen stabilizes intracellular activated Notch by targeting the Sel10 protein. Proc. Natl. Acad. Sci. USA.

[64] Verma S.C., Borah S., Robertson E.S. (2004). Latency-associated nuclear antigen of Kaposi’s sarcoma-associated herpesvirus up-regulates transcription of human telomerase reverse transcriptase promoter through interaction with transcription factor Sp1. J. Virol..

[65] Kwun H.J., da Silva S.R., Qin H., Ferris R.L., Tan R., Chang Y., Moore P.S. (2011). The central repeat domain 1 of Kaposi’s sarcoma-associated herpesvirus (KSHV) latency associated-nuclear antigen 1 (LANA1) prevents cis MHC class I peptide presentation. Virology.

[66] Shamay M., Krithivas A., Zhang J., Hayward S.D. (2006). Recruitment of the de novo DNA methyltransferase Dnmt3a by Kaposi’s sarcoma-associated herpesvirus LANA. Proc. Natl. Acad. Sci. USA.

[67] Mesri M. (2014). Advances in Proteomic Technologies and Its Contribution to the Field of Cancer. Adv. Med..

[68] Suthaus J., Adam N., Grotzinger J., Scheller J., Rose-John S. (2011). Viral Interleukin-6: Structure, pathophysiology and strategies of neutralization. Eur. J. Cell Biol..

[69] Aoki Y., Narazaki M., Kishimoto T., Tosato G. (2001). Receptor engagement by viral interleukin-6 encoded by Kaposi sarcoma-associated herpesvirus. Blood.

[70] Chen D., Cousins E., Sandford G., Nicholas J. (2012). Human herpesvirus 8 viral interleukin-6 interacts with splice variant 2 of vitamin K epoxide reductase complex subunit 1. J. Virol..

[71] Li Q., Chen D., Xiang Q., Nicholas J. (2019). Insulin-Like Growth Factor 2 Receptor Expression Is Promoted by Human Herpesvirus 8-Encoded Interleukin-6 and Contributes to Viral Latency and Productive Replication. J. Virol..

[72] Aoki Y., Jaffe E.S., Chang Y., Jones K., Teruya-Feldstein J., Moore P.S., Tosato G. (1999). Angiogenesis and hematopoiesis induced by Kaposi’s sarcoma-associated herpesvirus-encoded interleukin-6. Blood.

[73] Fullwood R.A., Low G.M., Chase E.P., Grasley M., Beal S.S., McCrary I.M., Daniels C.W., Ingersoll K., Berges B.K. (2018). The Kaposi’s sarcoma-associated herpesvirus viral interleukin 6 gene affects metastasis and expression of B cell markers in a murine xenograft model. PLoS ONE.

[74] Li W., Wang Q., Qi X., Guo Y., Lu H., Chen Y., Lu Z., Yan Q., Zhu X., Jung J.U. (2020). Viral interleukin-6 encoded by an oncogenic virus promotes angiogenesis and cellular transformation by enhancing STAT3-mediated epigenetic silencing of caveolin 1. Oncogene.

[75] Zhou J., Wang T., Zhang H., Liu J., Wei P., Xu R., Yan Q., Chen G., Li W., Gao S.J. (2024). KSHV vIL-6 promotes SIRT3-induced deacetylation of SERBP1 to inhibit ferroptosis and enhance cellular transformation by inducing lipoyltransferase 2 mRNA degradation. PLoS Pathog..

[76] Zhou F., Xue M., Qin D., Zhu X., Wang C., Zhu J., Hao T., Cheng L., Chen X., Bai Z. (2013). HIV-1 Tat promotes Kaposi’s sarcoma-associated herpesvirus (KSHV) vIL-6-induced angiogenesis and tumorigenesis by regulating PI3K/PTEN/AKT/GSK-3beta signaling pathway. PLoS ONE.

[77] Zhu X., Guo Y., Yao S., Yan Q., Xue M., Hao T., Zhou F., Zhu J., Qin D., Lu C. (2014). Synergy between Kaposi’s sarcoma-associated herpesvirus (KSHV) vIL-6 and HIV-1 Nef protein in promotion of angiogenesis and oncogenesis: Role of the AKT signaling pathway. Oncogene.

[78] Jung J.U., Stager M., Desrosiers R.C. (1994). Virus-encoded cyclin. Mol. Cell. Biol..

[79] Li M., Lee H., Yoon D.W., Albrecht J.C., Fleckenstein B., Neipel F., Jung J.U. (1997). Kaposi’s sarcoma-associated herpesvirus encodes a functional cyclin. J. Virol..

[80] Carbone A., Gloghini A., Bontempo D., Monini P., Tirelli U., Volpe R., Browning P.J., Gaidano G. (2000). Proliferation in HHV-8-positive primary effusion lymphomas is associated with expression of HHV-8 cyclin but independent of p27(kip1). Am. J. Pathol..

[81] Verschuren E.W., Jones N., Evan G.I. (2004). The cell cycle and how it is steered by Kaposi’s sarcoma-associated herpesvirus cyclin. J. Gen. Virol..

[82] Van Dross R., Yao S., Asad S., Westlake G., Mays D.J., Barquero L., Duell S., Pietenpol J.A., Browning P.J. (2005). Constitutively active K-cyclin/cdk6 kinase in Kaposi sarcoma-associated herpesvirus-infected cells. J. Natl. Cancer Inst..

[83] Zhi H., Zahoor M.A., Shudofsky A.M., Giam C.Z. (2015). KSHV vCyclin counters the senescence/G1 arrest response triggered by NF-kappaB hyperactivation. Oncogene.

[84] DiMaio T.A., Vogt D.T., Lagunoff M. (2020). KSHV requires vCyclin to overcome replicative senescence in primary human lymphatic endothelial cells. PLoS Pathog..

[85] Davis D.A., Shrestha P., Yarchoan R. (2023). Hypoxia and hypoxia-inducible factors in Kaposi sarcoma-associated herpesvirus infection and disease pathogenesis. J. Med. Virol..

[86] Kumar Singh R., Pei Y., Bose D., Lamplugh Z.L., Sun K., Yuan Y., Lieberman P., You J., Robertson E.S. (2021). KSHV-encoded vCyclin can modulate HIF1alpha levels to promote DNA replication in hypoxia. eLife.

[87] Wan Q., Tavakoli L., Wang T.Y., Tucker A.J., Zhou R., Liu Q., Feng S., Choi D., He Z., Gack M.U. (2024). Hijacking of nucleotide biosynthesis and deamidation-mediated glycolysis by an oncogenic herpesvirus. Nat. Commun..

[88] Chaudhary P.M., Jasmin A., Eby M.T., Hood L. (1999). Modulation of the NF-kappa B pathway by virally encoded death effector domains-containing proteins. Oncogene.

[89] Jacobs S.R., Damania B. (2011). The viral interferon regulatory factors of KSHV: Immunosuppressors or oncogenes?. Front. Immunol..

[90] Ballon G., Chen K., Perez R., Tam W., Cesarman E. (2011). Kaposi sarcoma herpesvirus (KSHV) vFLIP oncoprotein induces B cell transdifferentiation and tumorigenesis in mice. J. Clin. Investig..

[91] Field N., Low W., Daniels M., Howell S., Daviet L., Boshoff C., Collins M. (2003). KSHV vFLIP binds to IKK-gamma to activate IKK. J. Cell Sci..

[92] Hunte R., Alonso P., Thomas R., Bazile C.A., Ramos J.C., van der Weyden L., Dominguez-Bendala J., Khan W.N., Shembade N. (2018). CADM1 is essential for KSHV-encoded vGPCR-and vFLIP-mediated chronic NF-kappaB activation. PLoS Pathog..

[93] Nakayama R., Ueno Y., Ueda K., Honda T. (2019). Latent infection with Kaposi’s sarcoma-associated herpesvirus enhances retrotransposition of long interspersed element-1. Oncogene.

[94] Chen L., Ding L., Wang X., Huang Y., Gao S.J. (2024). Activation of glucocorticoid receptor signaling inhibits KSHV-induced inflammation and tumorigenesis. mBio.

[95] Russo J.J., Bohenzky R.A., Chien M.C., Chen J., Yan M., Maddalena D., Parry J.P., Peruzzi D., Edelman I.S., Chang Y. (1996). Nucleotide sequence of the Kaposi sarcoma-associated herpesvirus (HHV8). Proc. Natl. Acad. Sci. USA.

[96] Rivas C., Thlick A.E., Parravicini C., Moore P.S., Chang Y. (2001). Kaposi’s sarcoma-associated herpesvirus LANA2 is a B-cell-specific latent viral protein that inhibits p53. J. Virol..

[97] Baresova P., Musilova J., Pitha P.M., Lubyova B. (2014). p53 tumor suppressor protein stability and transcriptional activity are targeted by Kaposi’s sarcoma-associated herpesvirus-encoded viral interferon regulatory factor 3. Mol. Cell. Biol..

[98] Baresova P., Pitha P.M., Lubyova B. (2012). Kaposi sarcoma-associated herpesvirus vIRF-3 protein binds to F-box of Skp2 protein and acts as a regulator of c-Myc protein function and stability. J. Biol. Chem..

[99] Pfeffer S., Sewer A., Lagos-Quintana M., Sheridan R., Sander C., Grasser F.A., van Dyk L.F., Ho C.K., Shuman S., Chien M. (2005). Identification of microRNAs of the herpesvirus family. Nat. Methods.

[100] Cai X., Lu S., Zhang Z., Gonzalez C.M., Damania B., Cullen B.R. (2005). Kaposi’s sarcoma-associated herpesvirus expresses an array of viral microRNAs in latently infected cells. Proc. Natl. Acad. Sci. USA.

[101] Samols M.A., Hu J., Skalsky R.L., Renne R. (2005). Cloning and identification of a microRNA cluster within the latency-associated region of Kaposi’s sarcoma-associated herpesvirus. J. Virol..

[102] Yogev O., Henderson S., Hayes M.J., Marelli S.S., Ofir-Birin Y., Regev-Rudzki N., Herrero J., Enver T. (2017). Herpesviruses shape tumour microenvironment through exosomal transfer of viral microRNAs. PLoS Pathog..

[103] Gottwein E., Corcoran D.L., Mukherjee N., Skalsky R.L., Hafner M., Nusbaum J.D., Shamulailatpam P., Love C.L., Dave S.S., Tuschl T. (2011). Viral microRNA targetome of KSHV-infected primary effusion lymphoma cell lines. Cell Host Microbe.

[104] Bartel D.P. (2009). MicroRNAs: Target recognition and regulatory functions. Cell.

[105] Bellare P., Ganem D. (2009). Regulation of KSHV lytic switch protein expression by a virus-encoded microRNA: An evolutionary adaptation that fine-tunes lytic reactivation. Cell Host Microbe.

[106] Lu C.C., Li Z., Chu C.Y., Feng J., Feng J., Sun R., Rana T.M. (2010). MicroRNAs encoded by Kaposi’s sarcoma-associated herpesvirus regulate viral life cycle. EMBO Rep..

[107] Lei X., Bai Z., Ye F., Xie J., Kim C.G., Huang Y., Gao S.J. (2010). Regulation of NF-kappaB inhibitor IkappaBalpha and viral replication by a KSHV microRNA. Nat. Cell Biol..

[108] Liang D., Gao Y., Lin X., He Z., Zhao Q., Deng Q., Lan K. (2011). A human herpesvirus miRNA attenuates interferon signaling and contributes to maintenance of viral latency by targeting IKKepsilon. Cell Res..

[109] Liang Z., Qin Z., Riker A.I., Xi Y. (2020). CRISPR/Cas9 ablating viral microRNA promotes lytic reactivation of Kaposi’s sarcoma-associated herpesvirus. Biochem. Biophys. Res. Commun..

[110] Gottwein E., Mukherjee N., Sachse C., Frenzel C., Majoros W.H., Chi J.T., Braich R., Manoharan M., Soutschek J., Ohler U. (2007). A viral microRNA functions as an orthologue of cellular miR-155. Nature.

[111] Skalsky R.L., Samols M.A., Plaisance K.B., Boss I.W., Riva A., Lopez M.C., Baker H.V., Renne R. (2007). Kaposi’s sarcoma-associated herpesvirus encodes an ortholog of miR-155. J. Virol..

[112] Qin Z., Freitas E., Sullivan R., Mohan S., Bacelieri R., Branch D., Romano M., Kearney P., Oates J., Plaisance K. (2010). Upregulation of xCT by KSHV-encoded microRNAs facilitates KSHV dissemination and persistence in an environment of oxidative stress. PLoS Pathog..

[113] Lei X., Zhu Y., Jones T., Bai Z., Huang Y., Gao S.J. (2012). A Kaposi’s sarcoma-associated herpesvirus microRNA and its variants target the transforming growth factor beta pathway to promote cell survival. J. Virol..

[114] Suffert G., Malterer G., Hausser J., Viiliainen J., Fender A., Contrant M., Ivacevic T., Benes V., Gros F., Voinnet O. (2011). Kaposi’s sarcoma herpesvirus microRNAs target caspase 3 and regulate apoptosis. PLoS Pathog..

[115] Gottwein E., Cullen B.R. (2010). A human herpesvirus microRNA inhibits p21 expression and attenuates p21-mediated cell cycle arrest. J. Virol..

[116] Qin Z., DeFee M., Isaacs J.S., Parsons C. (2010). Extracellular Hsp90 serves as a co-factor for MAPK activation and latent viral gene expression during de novo infection by KSHV. Virology.

[117] Nayar U., Lu P., Goldstein R.L., Vider J., Ballon G., Rodina A., Taldone T., Erdjument-Bromage H., Chomet M., Blasberg R. (2013). Targeting the Hsp90-associated viral oncoproteome in gammaherpesvirus-associated malignancies. Blood.

[118] Nayar U., Sadek J., Reichel J., Hernandez-Hopkins D., Akar G., Barelli P.J., Sahai M.A., Zhou H., Totonchy J., Jayabalan D. (2017). Identification of a nucleoside analog active against adenosine kinase-expressing plasma cell malignancies. J. Clin. Investig..

[119] White M.C., Wong J.P., Damania B. (2024). Inhibition of NEK2 Promotes Chemosensitivity and Reduces KSHV-positive Primary Effusion Lymphoma Burden. Cancer Res. Commun..

[120] Sin S.H., Roy D., Wang L., Staudt M.R., Fakhari F.D., Patel D.D., Henry D., Harrington W.J., Damania B.A., Dittmer D.P. (2007). Rapamycin is efficacious against primary effusion lymphoma (PEL) cell lines in vivo by inhibiting autocrine signaling. Blood.

[121] Bhatt A.P., Bhende P.M., Sin S.H., Roy D., Dittmer D.P., Damania B. (2010). Dual inhibition of PI3K and mTOR inhibits autocrine and paracrine proliferative loops in PI3K/Akt/mTOR-addicted lymphomas. Blood.

[122] Li X., Wang F., Zhang X., Sun Q., Kuang E. (2023). Suppression of KSHV lytic replication and primary effusion lymphoma by selective RNF5 inhibition. PLoS Pathog..

[123] Moriguchi M., Watanabe T., Kadota A., Fujimuro M. (2019). Capsaicin Induces Apoptosis in KSHV-Positive Primary Effusion Lymphoma by Suppressing ERK and p38 MAPK Signaling and IL-6 Expression. Front. Oncol..

[124] Shrestha P., Davis D.A., Veeranna R.P., Carey R.F., Viollet C., Yarchoan R. (2017). Hypoxia-inducible factor-1 alpha as a therapeutic target for primary effusion lymphoma. PLoS Pathog..

[125] Pria A.D., Pinato D., Roe J., Naresh K., Nelson M., Bower M. (2017). Relapse of HHV8-positive multicentric Castleman disease following rituximab-based therapy in HIV-positive patients. Blood.

[126] Uldrick T.S., Polizzotto M.N., Aleman K., Wyvill K.M., Marshall V., Whitby D., Wang V., Pittaluga S., O’Mahony D., Steinberg S.M. (2014). Rituximab plus liposomal doxorubicin in HIV-infected patients with KSHV-associated multicentric Castleman disease. Blood.

[127] Nishimoto N., Kanakura Y., Aozasa K., Johkoh T., Nakamura M., Nakano S., Nakano N., Ikeda Y., Sasaki T., Nishioka K. (2005). Humanized anti-interleukin-6 receptor antibody treatment of multicentric Castleman disease. Blood.

[128] Berwanger A., Stein S.C., Kany A.M., Gartner M., Loretz B., Lehr C.M., Hirsch A.K.H., Schulz T.F., Empting M. (2023). Disrupting Kaposi’s Sarcoma-Associated Herpesvirus (KSHV) Latent Replication with a Small Molecule Inhibitor. J. Med. Chem..

[129] Wu Y., Wang V., Yarchoan R. (2024). Pacritinib inhibits proliferation of primary effusion lymphoma cells and production of viral interleukin-6 induced cytokines. Sci. Rep..

